# Myxoinflammatory Fibroblastic Sarcoma of the Parotid Gland: First Case Report and Literature Review

**DOI:** 10.3389/fmed.2022.833822

**Published:** 2022-05-20

**Authors:** Changhong Wei, Xuejia Yang, Pingping Guo, Xiaoyu Chen, Chunjun Li, Jun Chen, Sufang Zhou

**Affiliations:** ^1^Department of Pathology, Guangxi Medical University Cancer Hospital, Nanning, China; ^2^National Center for International Research of Bio-Targeting Theranostics, Guangxi Key Laboratory of Bio-Targeting Theranostics, Collaborative Innovation Center for Targeting Tumor Diagnosis and Therapy, Guangxi Medical University, Nanning, China; ^3^Department of Ultrasound Imaging, Guangxi Medical University Cancer Hospital, Nanning, China; ^4^Department of Biochemistry and Molecular Biology, School of Pre-clinical Science, Guangxi Medical University, Nanning, China

**Keywords:** myxoinflammatory fibroblastic sarcoma, MIFS, soft tissue tumor, Vimentin, parotid gland

## Abstract

Myxoinflammatory fibroblastic sarcoma (MIFS) is a rare, low-grade malignant soft tissue tumor. Most of the previously reported cases about this tumor were diagnosed within the soft tissues. Here, we report a unique case of MIFS of the right parotid gland in a 39-year-old Chinese male. The tumor primarily consisted of an inflammatory area and a mucus-like area in a migratory distribution. A number of lymphocytes, neutrophils, viral-like cells with large nucleoli, and eosinophilic cytoplasm or Reed-Sternberg-like cells, as well as spindle cells and epithelial-like aberrant cells, were observed within the tumor. They were found to express Vimentin and CD10 protein and no other specific immunohistochemical markers. The various cytomorphology and immunohistochemical features of this tumor were highly consistent with MIFS found in other sites. Therefore, several leading pathologists ultimately confirmed the final diagnosis of MIFS in the right parotid gland after repeated deliberation. To our knowledge, this is the first case of MIFS occurring in the parotid gland. Thus, our study provides a novel basis for identifying the biological behavior of the tumor in MIFS and also allows us to better understand the pathology of this rare tumor.

## Introduction

Myxoinflammatory fibroblastic sarcoma (MIFS) is a rare, slow-growing, low-grade malignant soft tissue tumor (1). It was first described by Montgomery et al. (2), and several cases have since been reported around the world (3, 4). Clinically, MIFS is considered to be a superficial tumor found within the soft tissues, and generally manifests itself as a painless subcutaneous mass in the distal limb in adults. However, in recent years, a proportion of cases have been reported in several other non-extremity sites, such as the breast, buttocks, chest wall, and thighs (3, 5, 6). However, MIFS of the parotid gland has not been reported and described previously. In this study, we present a unique case of MIFS of the parotid gland in a Chinese patient with a characteristic histomorphology, consisting of inflammatory and mucin-like areas. It was characterized by the prominent morphological presentation of aberrant large cells, containing huge nucleoli with a typical immunophenotype (Vimentin protein positive), and other features that were consistent with the previously reported MIFS in other sites. The study was approved by the Institutional Review Board of the Cancer Hospital of Guangxi Medical University and was performed according to the principles of the “Declaration of Helsinki.” Written informed consent for this study was obtained from the patient prior to the study.

## Case Presentation

### Clinical Findings

**FIGURE 1:**
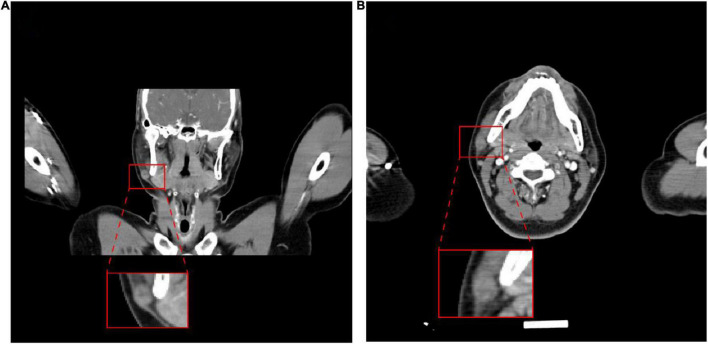
Images of the tumor. Computed tomography showed a nodule on the lower right side of the parotid gland. **(A)** Coronal plane; **(B)** transverse plane.

A 39-year-old male patient was diagnosed with a right submandibular mass in the outpatient clinic of our hospital 2 months ago. The physical examination showed that the neck curvature and mobility were normal, no lateral curvature was noticed, and the right lower jaw could reach the size of a 2 × 1-cm mass. Additionally, the mass was hard, had unclear boundary and blunt edge with poor activity, but no other abnormality was observed in other parts. After the patient was admitted to the hospital, the clinician used a fine needle for aspiration of cells from the tumor. The pathological examination further revealed that the nuclear atypical cells were visible, and the malignant possibility was not excluded. The ultrasound of the neck showed a substantial lesion of the right parotid gland, and the CT examination displayed a small nodule below the parotid gland (Figures 1A,B), which was initially considered as a benign tumor or tumor-like lesion of the parotid gland. His laboratory tests for the blood routine analysis, liver function, renal function, blood sugar, and thyroid function were found to be within the normal range. His HIV, HBsAg, and HCV serology were also negative. Under the general anesthesia, the right parotid mass resection and right facial nerve anatomy were performed and, thereafter, submitted for the histopathological examination.

### Pathologic Findings

Moreover, on the gross specimen, there was a gray-red, gray-yellow nodular mass within the parotid gland tissue, the size was 1.8 × 1.5 × 1 cm, and there was a complete capsule. The cut surface was grayish-yellow, gray, and white, soft, mucoid, and exhibited no hemorrhagic necrosis.

**FIGURE 2:**
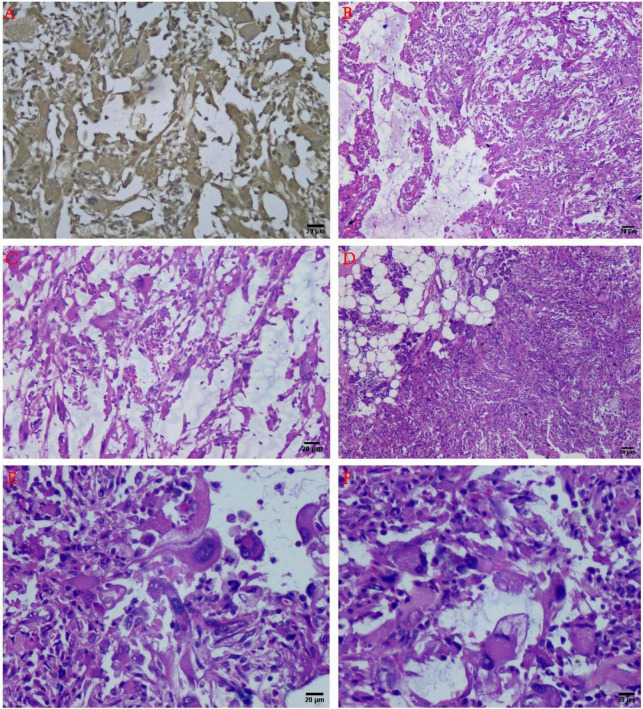
Histology and immunohistochemistry of the tumor. **(A)** Immunohistochemical results showed Vimentin diffuse positive (+) (IHC staining, ×100). **(B)** The tumor consisted of the mucous area and the fibrous area with inflammatory cell infiltration in the mucus-like area, and the various fusiform cells can be connected to each other to form a network structure (H&E staining, ×100). **(C)** At the junction of the mucous area and the fiber area, inflammatory cell infiltration could be observed (H&E staining, ×100). **(D)** The tumor can be infiltrated into the parotid tissue (H&E staining, ×100). **(E)** Large malformed cells with large nucleoli can be noted (H&E staining, ×200). **(F)** The “inclusion body” nucleolus resembling Reed-Sternberg cells has been recognized as one of the characteristics of mucinous inflammatory fibroblastic sarcoma (H&E staining, ×200).

**FIGURE 3:**
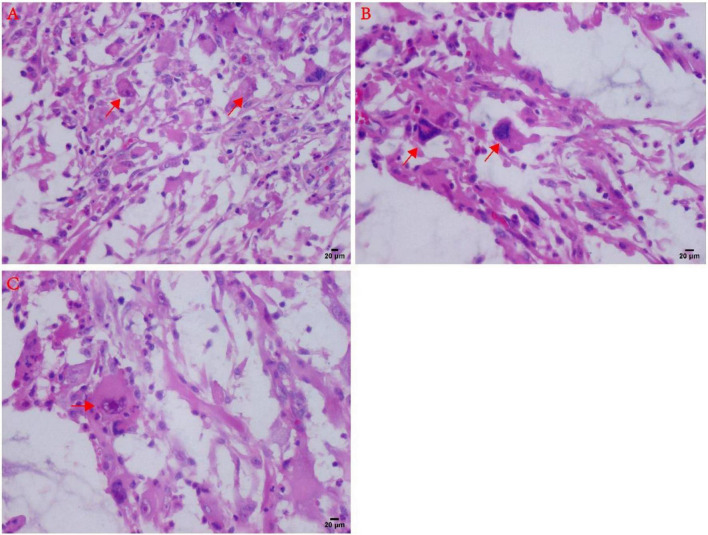
Atypical microscopic features in MIFS. **(A)** Virus-like cells with abundant cytoplasm and nucleoli (arrows). **(B)** Large malformed cells with two enlarged nuclei shown by arrows; **(C)** R-S cell-like tumor cells with markedly large nucleoli (arrows).

Histopathological microscopy showed that the tumor consisted of the different inflammatory regions as well as mucin-like regions, and the inflammatory cells were mostly lymphocytes or neutrophils. The inflammatory regions showed large nucleoli, and the cytoplasm was eosinophilic with viral-like cells or Reed-Sternberg-like cells, and it exhibited different degrees of fibrosis. The mucus region was primarily composed of spindle cells and tissue-like or epithelial-like malformed cells. The interstitial was hyaline degeneration, and small amounts of scattered multinucleated giant cells were visible in the lesion. Transitional distribution of inflammatory and mucous areas, visible large cells of nucleoli, and mitotic figures were not commonly observed, but mildly shaped spindle cells were seen in the inflammatory area. A part of the area was characterized by mucus and fibrotic regional composition, accompanied by lymphocyte or neutrophil infiltration. The most characteristic morphological manifestations were the presence of the large cells with huge nucleoli, with interstitial inflammatory cell reactions. Moreover, visible in the mucus area were the pseudo-fat cells, spindle cells involving adipose tissue, whereas the phagocytic cells can also be seen in some areas (Figures 2B–F, 3).

### Immunohistochemical Findings

**FIGURE 4:**
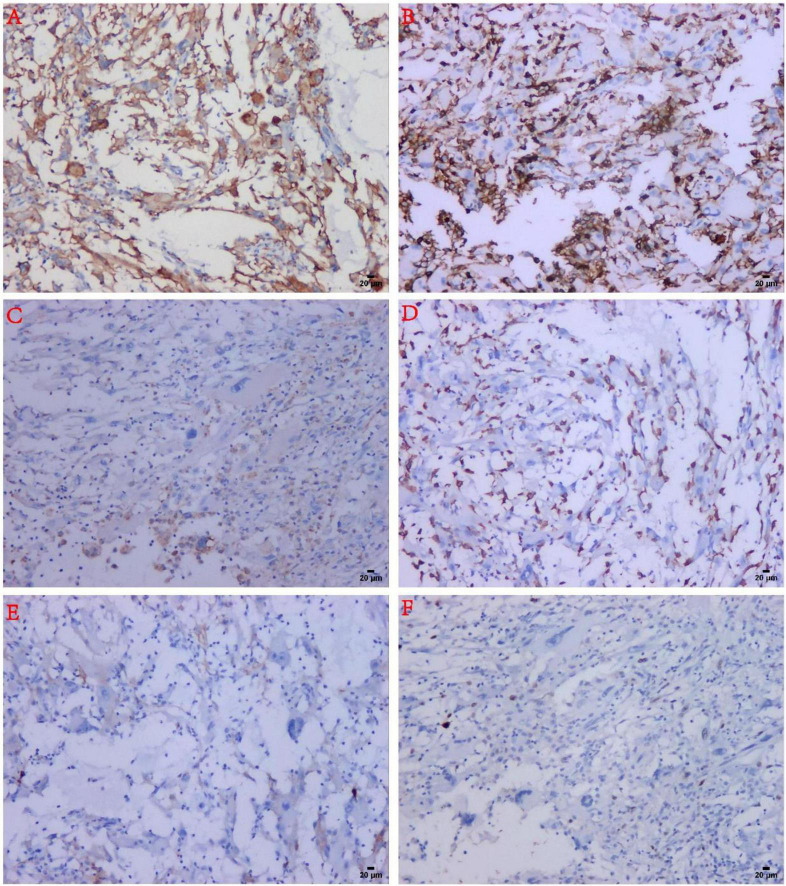
Immunohistochemical findings in MIFS. **(A)** The tumor spindle cells and malformed cells were strongly positive for CD10. **(B)** Infiltrated inflammatory cells were strongly positive for LCA. **(C)** The reactive histiocytes were positive for CD68. **(D)** Reactive histiocytes were positive for CD163. **(E)** The tumor cells were negative for D2-40. **(F)** The tumor cells expressed low-proliferative Ki-67 (about 10%).

Immunohistochemistry revealed that the tumor cells expressed Vimentin and CD10 (diffuse positive) (Figures 2A, 4A), lymphocytes expressed LCA, macrophages expressed CD163 and CD68, Ki-67 index was 10%, CK, SMA, P63, CD34, CD1a, D2-40, and S-100 were negative (Table 1 and Figure 4).

**TABLE 1:** A summary of the primary antibodies used and the results of immunohistochemistry.

Antibody	Source	Dilution	Result
CKpan	Maixin, Fuzhou, China	1:100	−
Vimentin	Maixin, Fuzhou, China	1:150	+++
LCA	Maixin, Fuzhou, China	1:100	+
CD68	Maixin, Fuzhou, China	1:100	+
Ki-67	Maixin, Fuzhou, China	1:200	+ (10%)
SMA	Maixin, Fuzhou, China	1:100	−
P63	Maixin, Fuzhou, China	1:100	−
CD34	Maixin, Fuzhou, China	1:100	−
Calponin	Maixin, Fuzhou, China	1:100	−
SMMHC	Maixin, Fuzhou, China	1:100	−
CD1a	Maixin, Fuzhou, China	1:100	−
S-100	Maixin, Fuzhou, China	1:100	−
CD163	Maixin, Fuzhou, China	1:100	+
CD10	Maixin, Fuzhou, China	1:100	+
D2-40	Maixin, Fuzhou, China	1:100	−

According to tumor imaging, microscopic histomorphology, and comprehensive immunohistochemical analysis, the authors finally diagnosed the tumor as parotid myxoinflammatory fibroblastic sarcoma. The patient is currently in good condition and is undergoing regular follow-up in our hospital, and no recurrence or metastasis has been found.

## Discussion

Based on the previously published review of domestic and foreign literature, this is the first report describing the occurrence of MIFS in the parotid gland. It has been reported that MIFS usually presents as a slow-growing painless mass, mostly in adults, and the peak age of the onset is 30–50 years old, the average age is about 40 years old. A small number of cases also occur in children or adolescents, and tumors primarily arise in the soft tissue location of the distal part of the limb (1, 7). In this study, we have summarized the clinicopathological features of MIFS reported in the literature in the rare sites (non-bone and soft tissue, since 1998) (Table 2) (6, 8, 9). Clinically, MIFS may be similar to benign lesions and can be often misdiagnosed as the slippery membrane inflammation, ganglion cysts, or giant cell tumor of the tendon sheath. CT or MRI usually presents as diffuse enhancement, lobulated, subcutaneous mass with non-specific magnetic resonance imaging features, thereby suggesting the presence of benign or malignant lesions (1).

**TABLE 2:** A summary of clinicopathological features of MIFS reported in the literature from the rare sites (non-bone and soft tissue, reviewed since 1998).

Author/year	Age/gender	Location	Pathological features	Treatment	Follow-up (mo)	Outcome
Current study	39/M	Parotid gland	The tumor consisted of inflammatory regions and mucin-like regions, the inflammatory cells were mostly lymphocytes or neutrophils. The most characteristic morphological manifestations are large cells with large nucleoli, with interstitial inflammatory cell reaction.	Surgery	28	No recurrence or metastasis
Jain et al. (8)	7-month-old infant	Eyeball	A mixed cellular infiltrate composed of numerous neutrophils, larger cells appeared to be binucleate (Reed-Sternberg-like or virocyte-like)	Resection	NA	NA
Auw-Haedrich et al. (6)	27/F	Iris	Tumor contained mucinous and cellular areas with high grade of polymorphy and a large variety of cell sizes and forms	Resection	15	No recurrence
Numminen et al. (9)	51/M	Nose	The tumor was heterogeneous in composition, with myxoid, relatively acellular areas alternating with cellular areas	Surgery	48	No recurrence or metastasis

*NA, not available; mo, month; F, female; M, male.*

The rich and diverse histological performance of MIFS is a major challenge in the diagnosis of this tumor (10). For example, the ratio of mucin-like areas, fibroinflammatory areas, and the cell area can effectively vary from case to case, and the number of cells with inclusion body-like nuclei is generally considered to be the major marker of MIFS. Another major challenge in diagnosis is the presence of severe inflammatory reactions, which are mediated primarily by the lymphocytes and lymphoid aggregates-infiltrated tumor cells. These may be mistaken for inflammatory processes or other malignant tumors, such as lymphoma mucinous fibrosarcoma (11). In the mucus background, the tumor spindle cells and epithelioid cells are usually connected to each other to form a complex network structure, and a large number of the neutrophils, macrophages, and plasma cells can be seen, but the mitotic figures and necrosis are not commonly observed in this tumor. Immunohistochemistry has been found to play a very limited role in the diagnosis of MIFS because it has no specific tumor markers (12).

In the present study, the main features for the diagnosis of MIFS included areas of solid spindle cells, alternating with myxoid foci with the diffuse infiltration of inflammatory cells. Unlike other previously reported cases, a random distribution of the larger atypical cells was seen in our case, including Reed-Sternberg cells, variants of virus-like cells with inclusion-like nucleoli, and large malformed cells. Immunohistochemistry has been widely used in MIFS, but no specific immunophenotype has been found. However, differential responsiveness to the various markers has been observed in the previous studies, including Vimentin, D2-40, CD68, bcl-1, CD10, and CD163 (3, 12). In the present study, we found consistent and strong immunoreactivity for Vimentin and CD10 in the tumor cells in this case. Moreover, another novel finding of our study was the low expression of the proliferative marker Ki-67, and the most characteristic phenomenon was that, in the large malformed cells and R-S like-cells, Ki-67 was almost absent. These pieces of evidence suggested that these atypical cells were the degenerative tumor cells that were biologically inactive.

The cytogenetic analysis of MIFS further revealed complex genetic abnormalities (13), including a circular chromosome formed on the Chromosome 3, a translocation of chromosome t (1:10) (p22: q24), rearrangement of *TGFBR3* and *MGEA5* genes (14). Some other cases showed *TGFBR3* and *MGEA5* gene rearrangement, which led to an upregulation of NPM3 and FGF8, accompanied by amplification of the *VGLL3* locus (10, 15).

Most cases of MIFS have demonstrated low malignant potential. However, 22–67% of cases can potentially recur, and only a few cases can metastasize (3), but high-grade MIFS may lead to more adverse progression (16). The best treatment for MIFS is complete surgical resection to ensure that the incisal margin is negative; however, the beneficial impact of radiotherapy and chemotherapy remains unclear (17). Our patient remained in good condition after 7 months of follow-up, and no recurrence or metastasis was observed.

## Conclusion

In summary, we report that the first case of MIFS was detected in the parotid gland. However, due to its complex histological morphology and inflammatory background, we need to distinguish it from other similar lesions, such as inflammatory myofibroblastic sarcoma and myxofibrosarcomas, to facilitate accurate diagnosis. Although no specific immunohistochemical or molecular markers are available for the diagnosis of this tumor. In addition to describing the classical histological features in this study. Our results supported the use of a distinct panel of immunohistochemical markers, including CD10 and Vimentin, might be helpful support for MIFS diagnostics in many equivocal cases. At present, the diagnosis of MIFS is still mainly based on clinicopathological diagnosis, which needs to be combined with the appropriate clinical environment and characteristic histomorphological findings to significantly improve the treatment outcome.

## Data Availability Statement

The raw data supporting the conclusions of this article will be made available by the authors, without undue reservation.

## Ethics Statement

The studies involving human participants were reviewed and approved by the Medical Ethics Committee of the Affiliated Cancer Hospital of Guangxi Medical University. The patients/participants provided their written informed consent to participate in this study. Written informed consent was obtained from the individual(s) for the publication of any potentially identifiable images or data included in this article.

## Author Contributions

JC and SZ read and approved the final manuscript. CW performed the writing of the manuscript. XY and PG organized the material and helped with the analysis. XC carried out experiments. CL and JC performed the pathological analysis and diagnosed the patient. All authors wrote and revised the manuscript.

## Conflict of Interest

The authors declare that the research was conducted in the absence of any commercial or financial relationships that could be construed as a potential conflict of interest.

## Publisher’s Note

All claims expressed in this article are solely those of the authors and do not necessarily represent those of their affiliated organizations, or those of the publisher, the editors and the reviewers. Any product that may be evaluated in this article, or claim that may be made by its manufacturer, is not guaranteed or endorsed by the publisher.
